# Case Report: Hepatoblastoma with spindle cell sarcomatous metastasis in a 14-year-old girl

**DOI:** 10.3389/fped.2026.1773950

**Published:** 2026-03-12

**Authors:** Yanting Zhang, Xuedi Yu, Junjie Ge, Jingjing Wang, Chuanfeng Bai, Suyi Kang, Jingfu Wang

**Affiliations:** 1 Department of Pediatric Oncology, Shandong Cancer Hospital and Institute, Shandong First Medical University and Shandong Academy of Medical Sciences, Jinan, Shandong, China; 2 Department of Pathology, Shandong Cancer Hospital and Institute, Shandong First Medical University and Shandong Academy of Medical Sciences, Jinan, Shandong, China

**Keywords:** alpha-fetoprotein, chemotherapy, hepatoblastoma, metastasis, spindle cell sarcoma

## Abstract

Hepatoblastoma (HB) is the most common pediatric liver malignancy and occurs predominantly in children younger than 5 years of age. We report the case of a 14-year-old girl who was diagnosed with mixed epithelial–mesenchymal hepatoblastoma without any metastases. Initial evaluation revealed a markedly elevated serum alpha-fetoprotein level (7,800 ng/mL) and a large hepatic mass (17.3 cm × 18.4 cm × 8.5 cm) in the left liver lobe on contrast-enhanced CT. The patient underwent surgical resection, and pathological examination confirmed mixed epithelial–mesenchymal HB. Postoperative chemotherapy (consisting of cisplatin, doxorubicin, and 5-FU) normalized serum alpha-fetoprotein levels; however, skull metastasis developed during treatment. Histopathological analysis of the metastatic lesion revealed spindle cell sarcoma with decreased GPC-3/Hepa expression and elevated CD34/Ki-67 expression. Multiple chemotherapy regimens (ifosfamide, carboplatin, and etoposide; and doxorubicin, vincristine, cyclophosphamide, and cisplatin) demonstrated limited efficacy. Subsequent treatment with alternating albumin-paclitaxel, gemcitabine, ifosfamide, and etoposide/cyclophosphamide, irinotecan, and vincristine chemotherapy combined with anlotinib and cranial radiotherapy achieved disease stabilization, with no subsequent progression observed during follow-up. This case highlights the aggressive nature and chemoresistance of the mesenchymal components of HB, emphasizing the need for novel therapeutic approaches that incorporate targeted agents.

## 1 Introduction

Hepatoblastoma (HB) is a rare malignant liver tumor that predominantly affects young children and is typically diagnosed in those under 3 years of age. It often presents as a rapidly growing hepatic mass, leading to symptoms such as abdominal distension, pain, jaundice, and elevated alpha-fetoprotein (AFP) levels (1–3). AFP, typically produced by the fetal liver and yolk sac, with normal levels declining after birth, is considered a useful clinical marker for diagnosis; however, approximately 10% of patients with HB do not exhibit elevated AFP levels (4, 5). The lung is the most common distant metastatic organ for HB, with 10%–20% of children presenting with lung metastases at diagnosis (6).

According to the International Pediatric Liver Tumor Consensus Classification, HB is morphologically classified as either entirely epithelial or mixed (epithelial and mesenchymal) types (6). HB with mesenchymal differentiation is associated with a more aggressive clinical course and a higher risk of metastasis compared to those composed purely of epithelial components. These mesenchymal elements, when present, can also contribute to the tumor's potential for local invasion and distant spread, posing significant challenges in clinical management.

The SIOPEL initiative, which incorporates cisplatin-based neoadjuvant chemotherapy in combination with surgery and postoperative chemotherapy, has shown a significant improvement in patient prognosis (2). However, optimal treatment strategies for high-risk pediatric patients with extrahepatic involvement or distant metastases remain to be explored. We present the case of a 14-year-old girl who was diagnosed with mixed epithelial–mesenchymal HB. During adjuvant chemotherapy, the patient developed skull metastases, which were confirmed to consist of mesenchymal components histologically.

## 2 Case report

A 14-year-old girl who was previously in good health presented with left shoulder pain one day. She had no family history of cancer, no history of hepatitis B virus infection or alcohol abuse, and was mentally sound. Abdominal ultrasonography revealed a large hepatic mass, and contrast-enhanced computed tomography confirmed a mass measuring approximately 17.3 cm × 18.4 cm × 8.5 cm in the left liver lobe, along with slightly enlarged retroperitoneal lymph nodes. At initial presentation, the serum AFP level was 7,800 ng/mL (reference range: 0–15 ng/mL).

**Figure 1:**
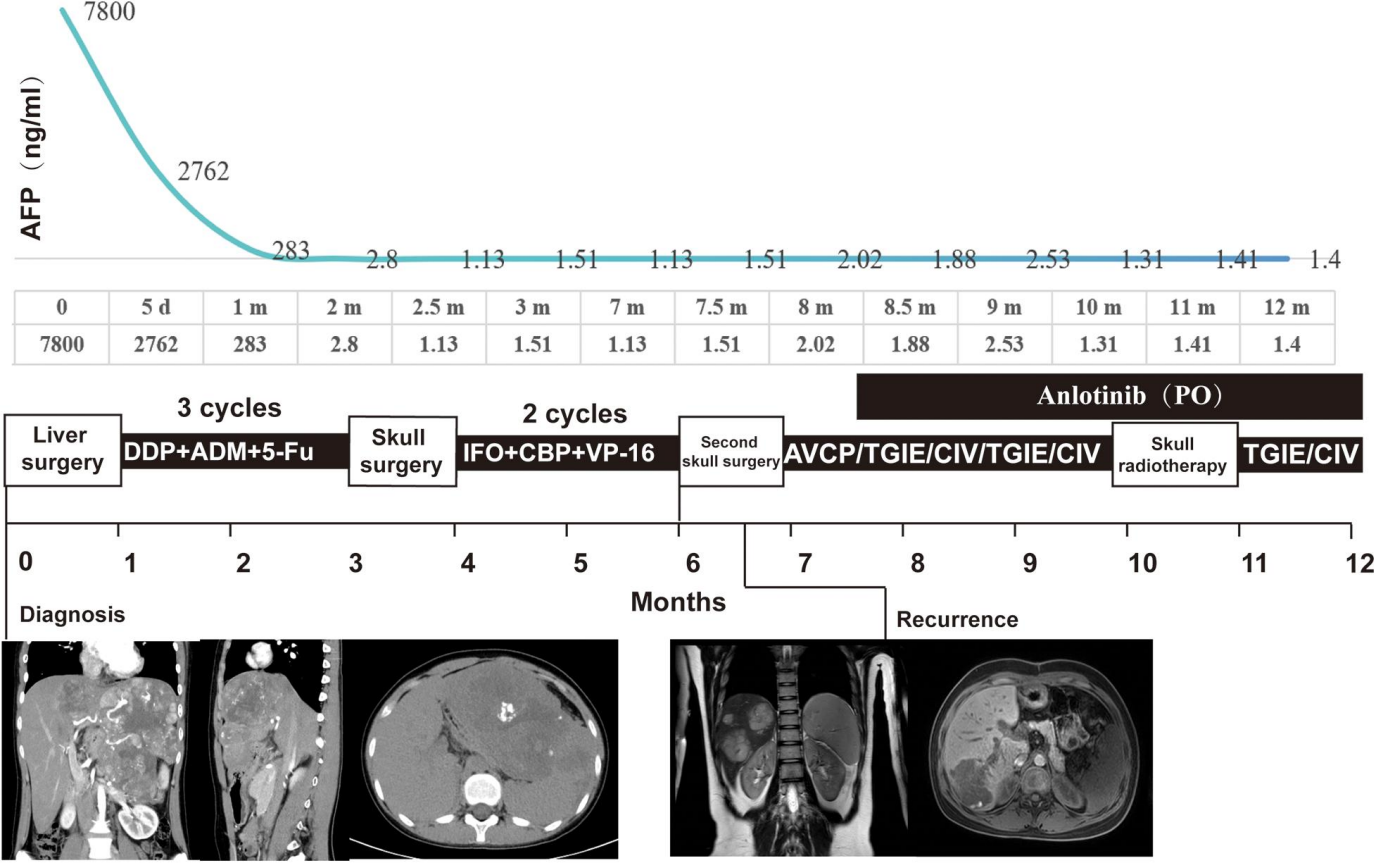
Clinical management trajectory, serum AFP dynamics, and corresponding imaging findings of the patient with HB over 12 months. The upper section illustrated the temporal evolution of AFP levels, with specific values recorded at key time points from diagnosis (0) to 12 months. The middle timeline detailed the sequential therapeutic interventions, including liver surgery followed by three cycles of chemotherapy(cisplatin, doxorubicin, and fluorouracil), skull surgery with subsequent two cycles of chemotherapy(ifosfamide, carboplatin, and etoposide), a second liver surgery, administration of AVCP/TGIE/CIV regimens, oral anlotinib, and skull radiotherapy. The lower row of images provide visual documentation of abdominal pathologic changes, juxtaposing findings at initial diagnosis and at disease recurrence.

As shown in Figure 1, the patient underwent direct surgical resection of the liver tumor. Immunohistochemical (IHC) analysis of the primary hepatic lesion revealed the following findings: CK (AE1/AE3) (epithelial component +), CK7 (−), CK19 (−), Hepa-1 (epithelial component +), GPC-3 (epithelial component +), AFP (−), Arg-1 (focally weak +), CD34 (−), ERG (−), SALL4 (−), β-catenin (cell membrane+in the epithelial component), Syn (−), CgA (−), S-100 (−), desmin (−), CD117 (−), DOG1 (−), SMARCA4 (+), INI-1 (+), and Ki-67 (30%+). Combined with the immunohistochemical findings, the primary hepatic lesion was diagnosed as mixed epithelial–mesenchymal HB. The epithelial component was of fetal type (accounting for approximately 20%), and the mesenchymal component consisted of spindle cell sarcoma (accounting for approximately 80%), accompanied by necrosis.

**Figure 2:**
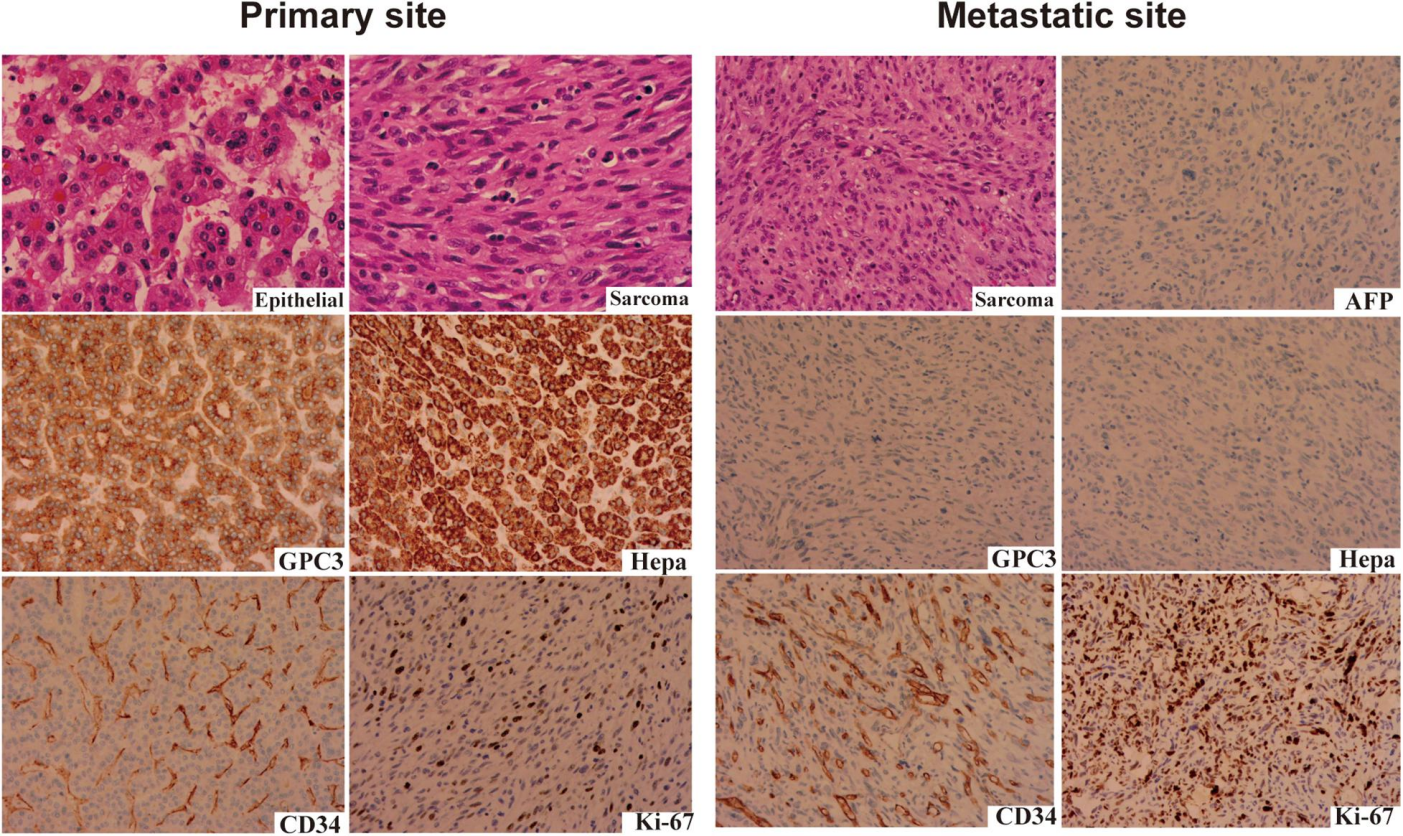
HE staining and IHC staining of the primary and metastatic sites tissues. The upper row presents HE sections: the primary site exhibits both epithelial and sarcomatous components, while the metastatic site shows a sarcomatous pattern. The middle and lower rows display IHC results for AFP, GPC-3, Hepa, CD34, and Ki-67.

The serum AFP level decreased from 7,800 ng/mL at diagnosis to 2,762 ng/mL 5 days after surgery. Initially, a cisplatin-based chemotherapy regimen is considered the standard treatment for mixed epithelial–mesenchymal HB; therefore, chemotherapy (cisplatin, doxorubicin, and fluorouracil) was started 3 weeks after surgery. After two cycles of chemotherapy, the AFP level decreased to 2.8 ng/mL and remained within the normal range. However, during the third cycle of chemotherapy, a mass (3.2 cm × 2.6 cm × 3.4 cm) in the skull was detected. Surgical resection of the skull mass was performed, and pathological examination confirmed metastatic HB composed only of spindle cell sarcoma components. Immunohistochemical examination revealed that (Figure 2), compared with those in the primary site, the metastatic tumor showed lower expression of GPC-3 and Hepa and higher expression of CD34 and Ki-67. The chemotherapy regimen was switched to the ICE regimen (ifosfamide, carboplatin, and etoposide), which is a first-line chemotherapy regimen for pediatric soft tissue sarcomas. Subsequently, this girl underwent a second surgical procedure due to poor wound healing and delayed recovery at the skull surgical site.

The patient was transferred to our hospital and treated with an AVCP chemotherapy regimen (doxorubicin, vincristine, cyclophosphamide, and cisplatin), which is considered a second-line chemotherapy regimen for soft tissue sarcomas. However, control of skull metastatic sites remained relatively poor. Therefore, we attempted salvage therapy using alternating cycles of TGIE (albumin-paclitaxel, gemcitabine, ifosfamide, etoposide) and CIV (cyclophosphamide, irinotecan, vincristine), in combination with oral anlotinib. Immunohistochemical analysis of the metastatic lesions showed a significant increase in CD34 expression (markedly upregulated compared with the primary tumor). In addition, the elevated Ki-67 proliferation index indicated active proliferation of tumor cells. Anti-angiogenic drugs can indirectly inhibit the growth of highly proliferative tumor cells by suppressing angiogenesis, thereby exerting a synergistic effect when combined with chemotherapy. After four cycles of TGIE and CIV regimens, the condition of the patient remained stable. Considering that the skull metastatic lesion of the patient was confined to the left parietal bone without intracranial invasion and presented as an isolated metastatic lesion, radiotherapy (4,500 Gy/15 fractions) was administered to control local tumor progression. Following radiotherapy, chemotherapy with the TGIE/CIV regimens was re-administered. The patient remains on treatment, with stable disease. We will continue to follow up to provide more robust evidence-based data on the long-term effectiveness of the treatment regimen and to further enhance the clinical reference value of this study.

## 3 Discussion

HB predominantly affects children aged between 6 months and 3 years of age, and most patients present with elevated levels of AFP at diagnosis (3). Approximately 15% of patients are found to have lung metastases at the time of diagnosis, while other metastases to other sites, such as the brain or bones, are relatively rare (7). Our case described involves a 14-year-old girl who presented with elevated AFP levels at diagnosis but without metastasis to other sites. However, during the third cycle of chemotherapy following resection of the primary site, the patient developed cranial bone metastasis, which is extremely uncommon. Therefore, despite the predominance of lung metastasis, this case highlights that metastases to other organs can occur, indicating the need for comprehensive monitoring and individualized treatment strategies for patients with HB.

HB can be categorized into two primary histological types: epithelial type and mixed epithelial–mesenchymal type (8). The epithelial type is further subdivided into several subtypes, such as embryonal, fetal, small cell undifferentiated (SCUD), cholangioblastic, and macrotrabecular patterns, occurring either individually or in combination. The mesenchymal component may comprise mature and immature fibrous tissue, osteoid or osteoid-like tissue, and hyaline cartilage, and in rare cases, tumors may also exhibit teratoid features.

Regarding the differential diagnosis of the primary lesion, we mainly distinguished it from hepatocellular carcinoma (HCC) and hepatic sarcoma. HCC in adolescents is relatively rare clinically and is mostly associated with underlying liver diseases, such as chronic hepatitis or liver cirrhosis. However, this patient had no history of hepatitis and no imaging findings of liver cirrhosis. In terms of serological indicators, AFP elevation is usually not significant in HCC patients, and abnormalities in tumor markers such as CEA and CA199 are often present. In contrast, the patient exhibited a markedly elevated AFP level (7,800 ng/mL), while the CEA and CA199 levels were within the normal range. Regarding histopathology and immunohistochemistry, HB expresses embryonic markers such as GPC-3 and Hepa, while HCC lacks CK19 expression. The primary lesion in this patient showed positive expression of both GPC-3 and Hepa, findings that are consistent with the characteristics of HB and further exclude the possibility of HCC. Hepatic sarcoma most commonly occurs in children and adolescents. Radiologically, it typically presents as a solid mass with ill-defined borders, frequent areas of necrosis and cystic changes, and heterogeneous enhancement on contrast-enhanced imaging. In contrast, the mass in this patient had relatively well-defined borders, no obvious necrosis or cystic changes, and an enhancement pattern distinct from that of hepatic sarcoma. Regarding immunohistochemistry, hepatic sarcomas mostly express mesenchymal markers, such as vimentin and SMA, but do not express HB-specific markers like AFP and GPC-3. In this case, the primary lesion demonstrated positive expression of AFP and GPC-3, while vimentin was only partially expressed within the mesenchymal component; this immunophenotype is inconsistent with that of hepatic sarcoma.

In addition, to confirm that the metastatic component originated from HB, we performed the following differential diagnoses. (1) Hematoxylin and eosin (HE) staining of the skull metastatic lesion showed a pure spindle cell sarcoma-like morphology, and the tumor cells were arranged in a disorganized, fascicular, or storiform pattern, with easily identifiable mitotic figures. Compared with the mesenchymal components of the primary tumor, the metastatic lesion was highly consistent in terms of cell morphology (spindle-shaped cells, increased nuclear-cytoplasmic ratio) and arrangement pattern (bundle-like). These findings suggest that the metastatic lesion originated from further malignant transformation and clonal proliferation of the mesenchymal components of the primary tumor, indicating a clear histological lineage relationship. (2) Based on the patient's medical history and imaging examinations, no evidence of a second primary malignancy was identified, thus excluding the possibility of skull metastasis originating from other types of sarcomas. (3) Immunohistochemical differentiation: Immunohistochemical staining of the skull metastatic lesion revealed the following results: CK (AE1/AE3) (−), CK7 (−), CK19 (−), EMA (−), hepatocyte (−), GPC-3 (focally +), AFP (+), catenin (−), FLI-1 (partially +), CD31 (−), CD34 (−), ERG (−), CD68 (−), desmin (−), myogenin (−), MyoD1 (−), S-100 (−), HMB45 (−), PLAP (−), SMARCA4/Brg1 (+), INI-1 (+), and Ki-67 (90%+).

Based on the immunohistochemical findings, the following differential diagnoses were considered: (1) Rhabdomyosarcoma: The diagnosis of rhabdomyosarcoma requires the expression of myogenic markers, including desmin, myogenin, and MyoD1. In this case, all three aforementioned markers were negative in the metastatic lesion [desmin (−), myogenin (−), and MyoD1 (−)], and no morphologic features of myoblast differentiation or cross-striations were identified. Thus, metastatic rhabdomyosarcoma was clearly excluded. (2) Angiosarcoma: Angiosarcoma expresses vascular endothelial markers such as CD31, CD34, and ERG. In this case, the metastatic lesion was negative for CD31 (−), CD34 (−), and ERG (−), and no vascular lumen-like structures were observed. These findings are inconsistent with the immunophenotypic and morphological characteristics of vascular tumors; therefore, angiosarcoma was ruled out. (3) Neurogenic sarcoma: Neurogenic sarcomas often express markers such as S-100 and GFAP. The metastatic lesion in this case was negative for S-100 (−), and no neurofibrillary bundles or palisading structures were observed. Therefore, metastatic neurogenic sarcoma was excluded.

Although the high-risk factors in the SIOPEL risk stratification protocol include only PRETEXT IV, AFP <100 ng/mL, extrahepatic disease, and tumor rupture (9, 10), a retrospective study has suggested that a mixed epithelial–mesenchymal histopathological pattern may also be associated with a poor prognosis (11). In this case, the pathological type of the primary tumor in the liver was identified as mixed epithelial–mesenchymal, which suggests that the patient may have been at increased risk for developing metastases. Immunohistochemical results showed that the expression of GPC-3 and Hepa (typical epithelial markers) in the metastatic lesions was significantly decreased, while the expression of CD34 (vascular endothelial marker) and Ki-67 (proliferation index) was increased. The transformation from the original mixed-type HB to a predominantly mesenchymal spindle cell sarcoma phenotype at the metastatic site could suggest clonal evolution or the selection of more aggressive subpopulations during the process of metastasis. Such changes highlight the heterogeneity of HB and underscore the complexity involved in predicting disease progression and response to therapy.

Previous literature has shown that AFP serves not only as an indicator for HB but also, to some extent, acts as a predictor of treatment efficacy and prognosis. After the patient underwent surgery and two cycles of chemotherapy, the AFP level decreased to within the normal range, indicating a favorable treatment outcome. However, in this case, the patient surprisingly developed skull metastases without a corresponding increase in AFP, which warrants our in-depth consideration. This case reminds us that AFP alone cannot serve as the sole indicator for monitoring therapeutic effect in mixed (epithelial and mesenchymal) HB and that a comprehensive assessment incorporating imaging examinations and additional molecular markers is necessary. Surgery and adjuvant chemotherapy have significantly improved the prognosis of patients with HB (12). The introduction of chemotherapy, particularly cisplatin-based chemotherapy regimens, has increased the 5-year survival rate of patients with HB to 80% (13). However, when HB progresses to metastatic spindle cell sarcoma, there is currently no clear treatment direction, posing a significant challenge for clinicians. The following treatment strategies may be required. First, intensive chemotherapy regimens are more effective against sarcoma. Second, anti-angiogenic drugs and other targeted therapeutics may enhance therapeutic efficacy by inhibiting tumor angiogenesis. Anlotinib is a multi-target tyrosine kinase inhibitor that blocks tumor neovascularization by inhibiting the vascular endothelial growth factor (VEGF)-mediated signaling pathway (14). It is considered one of the recommended targeted therapy agents for pediatric malignant solid tumors characterized by active angiogenesis (as indicated by high CD34 expression). In addition, for isolated metastatic lesions, surgical resection or radiotherapy may serve as an important salvage treatment (15).

During chemotherapy, the patient experienced adverse reactions such as nausea, vomiting, alopecia, and myelosuppression. The medical team closely monitored these complications, managed these adverse reactions through medications, and provided emotional support via psychological counseling. Consequently, the patient actively cooperated throughout the treatment process, maintained full confidence in the therapy, and completed all treatment courses as scheduled, which significantly contributed to disease stabilization.

This case report presents the diagnosis and treatment process of a rare mixed HB in an adolescent patient with cranial metastasis, highlighting its significant clinical implications. We found that (1) the mesenchymal components appear to be closely related to the enhanced tumor invasiveness and chemotherapy resistance; (2) the monitoring value of AFP in HB, which is dominated by mesenchymal components, is limited; (3) although bone metastasis is rare, it warrants special attention in mesenchymal HB; and (4) the efficacy of traditional chemotherapy regimens is limited, highlighting the need to explore comprehensive treatment plans including targeted therapy. Further studies are warranted to investigate the molecular drivers of sarcomatous transformation in HB, and these findings may guide the selection of targeted therapies. These findings provide valuable clinical reference for the diagnosis and treatment of similar cases and underscore the need to further explore the molecular mechanisms of HB to facilitate the development of more effective treatment strategies.

## Data Availability

The original contributions presented in the study are included in the article/Supplementary Material; further inquiries can be directed to the corresponding authors.

## References

[1] Cao Y Wu S Tang H. An update on diagnosis and treatment of hepatoblastoma. Biosci Trends. (2024) 17(6):445–57. 10.5582/bst.2023.01311 38143081

[2] Czauderna P Lopez-Terrada D Hiyama E Häberle B Malogolowkin MH Meyers RL. Hepatoblastoma state of the art: pathology, genetics, risk stratification, and chemotherapy. Curr Opin Pediatr. (2014) 26(1):19–28. 10.1097/MOP.0000000000000046 24322718

[3] Kong M Zhai Y Liu H Zhang S Chen S Li W Insights into the mechanisms of angiogenesis in hepatoblastoma. Front Cell Dev Biol*.* (2025) 13:1535339. 10.3389/fcell.2025.1535339 40438141 PMC12116456

[4] Ozer G Ozcan HN Ardicli B Kutluk T Oguz B Haliloglu M. Radiological and clinical signatures to differentiate hepatocellular carcinoma from hepatoblastoma in children older than 5 years of age: a feasibility study. Pediatr Radiol. (2025) 55(5):946–54. 10.1007/s00247-025-06190-w 39961817 PMC12065736

[5] Meyers RL Maibach R Hiyama E Häberle B Krailo M Rangaswami A Risk-stratified staging in paediatric hepatoblastoma: a unified analysis from the Children's Hepatic Tumors International Collaboration. Lancet Oncol. (2017) 18(1):122–31. 10.1016/S1470-2045(16)30598-8 27884679 PMC5650231

[6] Yang T Whitlock RS Vasudevan SA. Surgical management of hepatoblastoma and recent advances. Cancers (Basel). (2019) 11(12):1944. 10.3390/cancers11121944 31817219 PMC6966548

[7] Ho HT Mai TH Nguyen TX Nguyen K Pham NH Nguyen HS. Central hepatectomy in a 6-month-old child with hepatoblastoma following chemotherapy. Case Rep Oncol. (2021) 14(2):874–80. 10.1159/000516800 34267635 PMC8261264

[8] Jiao J Saxena R Morotti R. Hepatoblastoma: comprehensive review with recent updates. Adv Anat Pathol. (2025) 32:309–16. 10.1097/PAP.0000000000000495 40178831

[9] Meyers RL Rowland JR Krailo M Chen Z Katzenstein HM Malogolowkin MH. Predictive power of pretreatment prognostic factors in children with hepatoblastoma: a report from the Children's Oncology Group. Pediatr Blood Cancer. (2009) 53(6):1016–22. 10.1002/pbc.22088 19588519 PMC4408767

[10] Fuchs J Rydzynski J Von Schweinitz D Bode U Hecker H Weinel P Pretreatment prognostic factors and treatment results in children with hepatoblastoma: a report from the German Cooperative Pediatric Liver Tumor Study HB 94. Cancer. (2002) 95(1):172–82. 10.1002/cncr.10632 12115331

[11] Patil OG Prakash A Akshatha C Nargund A Cherian LB Balu S A clinicopathological study with risk-stratified staging of pediatric hepatoblastoma: a 5-year experience from a tertiary cancer center. Iran J Pathol. (2023) 18(2):165–72. 10.30699/IJP.2023.1972340.3005 37600579 PMC10439754

[12] Pagarigan A Mendoza P. Adult hepatoblastoma: making the challenging distinction from hepatocellular carcinoma. J Liver Cancer. (2023) 23(1):219–24. 10.17998/jlc.2023.02.24 37384033 PMC10202245

[13] Kong M Zhang S Ma X. Insights into the mechanisms of microRNAs in hepatoblastoma: from diagnosis to treatment. Precis Clin Med. (2025) 8(4):pbaf034. 10.1093/pcmedi/pbaf034 41393242 PMC12699220

[14] Shen G Zheng F Ren D Du F Dong Q Wang Z Anlotinib: a novel multi-targeting tyrosine kinase inhibitor in clinical development. J Hematol Oncol. (2018) 11(1):120. 10.1186/s13045-018-0664-7 30231931 PMC6146601

[15] Roohani S Wiltink LM Kaul D Spałek MJ Haas RL. Update on dosing and fractionation for neoadjuvant radiotherapy for localized soft tissue sarcoma. Curr Treat Options Oncol. (2024) 25(4):543–55. 10.1007/s11864-024-01188-2 38478330 PMC10997691

